# Investigation of tracer gas transport in a new numerical model of lung acini

**DOI:** 10.1007/s11517-022-02608-x

**Published:** 2022-07-06

**Authors:** Christoph Schmidt, Christoph Joppek, Frederik Trinkmann, Ralf Takors, Giorgio Cattaneo, Johannes Port

**Affiliations:** 1 Institute of Biomedical Engineering, University of Stuttgart, Seidenstraße 36, 70174 Stuttgart, Germany; 2 Pneumology and Critical Care Medicine, Thoraxklinik at University Hospital Heidelberg, Translational Lung Research Center Heidelberg (TLRC), Member of German Center for Lung Research (DZL), Heidelberg, Germany; 3 Department of Biomedical Informatics, Center for Preventive Medicine and Digital Health Baden-Württemberg (CPD-BW), University Medical Center Mannheim, Heidelberg University, Heidelberg, Germany; 4 Institute of Biochemical Engineering, University of Stuttgart, Stuttgart, Germany

**Keywords:** Acinar model, Single branch-point model, Gas distribution, Diffusion, Convection

## Abstract

Obstructive pulmonary diseases are associated with considerable morbidity. For an early diagnosis of these diseases, inert gas washouts can potentially be used. However, the complex interaction between lung anatomy and gas transport mechanisms complicates data analysis. In order to investigate this interaction, a numerical model, based on the finite difference method, consisting of two lung units connected in parallel, was developed to simulate the tracer gas transport within the human acinus. Firstly, the geometries of the units were varied and the diffusion coefficients (*D*) were kept constant. Secondly, *D* was changed and the geometry was kept constant. Furthermore, simple monoexponential growth functions were applied to evaluate the simulated data. In 109 of the 112 analyzed curves, monoexponential function matched simulated data with an accuracy of over 90%, potentially representing a suitable numerical tool to predict transport processes in further model extensions. For total flows greater than 5 × 10^−4^ ml/s, the exponential growth constants increased linearly with linear increasing flow to an accuracy of over 95%. The slopes of these linear trend lines of 1.23 µl^−1^ (*D* = 0.6 cm^2^/s), 1.69 µl^−1^ (*D* = 0.3 cm^2^/s), and 2.25 µl^−1^ (*D* = 0.1 cm^2^/s) indicated that gases with low *D* are more sensitive to changes in flows than gases with high *D*.

**Graphical abstract:**

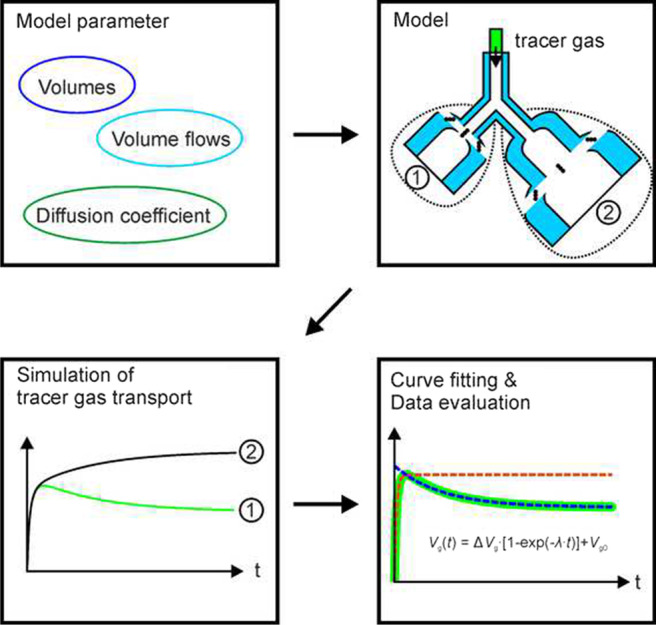

## Introduction

### Medical background

Obstructive lung diseases (OLD) such as bronchial asthma, chronic obstructive pulmonary disease (COPD), or cystic fibrosis are associated with considerable morbidity [1–4]. They result from multifactorial triggers, including but not limited to inflammatory processes, inhaled noxae, tissue remodelling, and genetic factors. COPD is among the most common diseases worldwide and represents the third leading cause of death [5]. OLD are characterized by an increased airway resistance especially in the peripheral lung, while also associated with an impaired compliance [6]. Both may lead to a dysfunction of the pulmonary ventilation [7]. In particular, morphological and functional changes in the small airways are mainly responsible for ventilation inhomogeneities (VI) impairing respiratory function [8]. Reproducible and reliable methods for the early detection of VI are essential for early detection, therapeutic monitoring, and thus the improvement of disease control and quality of life with OLD.

Small airway function comprises several domains and cannot be attributed to one single diagnostic test [9]. Commonly applied spirometry allows the assessment of several global lung function parameters mostly affected by central obstruction. It is therefore difficult to distinguish directly between central and peripheral airways [7]. In contrast, inert gas washouts (IGW) are potentially able to assess VI in the small airways. For example, an increase in VI was demonstrated in patients with bronchial asthma as compared to non-asthmatic controls. These differences also persist in asthmatic patients with normal spirometry and therefore add important diagnostic information [10].

In general, an endogenous or exogenous tracer gas is used to measure the local and global parameters of VI. These are applied in two different types of IGW, referred to as single breath washout (SBW) and multi breath washout (MBW). In SBW subjects, normal or medical air is breathed through a mouthpiece while wearing a nose clip until the inspiration and expiration curves reach a steady state. Then, the tracer gas is inhaled in the case that an exogenous tracer gas is used. Subsequently, it is exhaled for a breath either using a full vital capacity effort or tidal breathing, depending on the tracer gas used. Various tracer gases such as the exogenous tracer gases sulfur hexafluoride (SF_6_), helium (He), and the endogenous tracer gas nitrogen (N_2_) have been investigated in medical studies [11, 12]. In some areas such as cystic fibrosis, these tests have been adopted as a daily clinical routine. In other areas, such as bronchial asthma or COPD, their use is mostly limited to scientific settings. The concentration of the relevant tracer gases (N_2_, He, SF_6_) during the expiration phase is recorded and evaluated. In MBW, subjects breathe the tracer gas over several consecutive respiration cycles. Again, the concentration of the tracer gases decreasing under a pre-defined concentration during the subsequent expiration phase is used to calculate VI parameters [13–15].

Although these techniques can potentially provide complementary information to spirometry in the early stages of obstructive pulmonary diseases, the complexity of the measurement and interpretation, cost, availability, and lack of standardization currently limit clinical application [16].

### Numerical models

Numerical modelling is an important tool to investigate not only the transport of drugs and aerosols in the lungs [17, 18] but also the effect of bronchial tree inhomogeneity on local airflow pathways. This potentially supports the interpretation of the data recorded by IGW as well as the improvement of this technique, including treatment parameters and application of new gases.

In the past, several numerical lung models were conceived for the prediction of the effects of VI on the characteristic shapes in washout curves in healthy individual lungs [19–25]. In addition, models were presented which analyzed the mechanisms that lead to increased VI in OLD [26–28].

In principle, the works mentioned above followed the same approach by applying the washout procedure to the entire lungs (including all generations between trachea and alveolar sacks) [20, 21, 23, 24, 28] or to individual lung areas (acinus [25], generations 11–23 [26]) and analyzing the influence of the model parameters on the gas concentration profile during expiration. However, this approach has the disadvantage that the simulated curves, describing the gas transport within the lung airways, are very complex and, for instance, two or more exponential functions are used to evaluate them [29].

The objective of this work was to investigate the effect of geometry, flow rate, and diffusion properties on gas transport within the acinus in order to provide a mechanical context for OLD diagnostics. To achieve this objective, a parameter study was completed on a numerical model developed in-house. The numerical model was based on the finite difference method, in which the convective and diffusive molar fluxes were balanced in discrete compartments.

## Material and methods

Table 1 provides a brief and concise description of the procedure for this chapter.

**Table 1:** Overview of the sections in the “Material and methods” chapter

Method section	Short description
Geometrical assumptions	- The morphometric data derived by Weibel are the geometrical basis for the generations 17–23 (acinus) - For simplification, the diameter and lengths of the Weibel model were averaged through generations 17–23
Creation of a simple bronchial network	- *Simplification of Weibel’s model* by using the averaged parameter to create a model consisting of BrU0, BrU1, and BrU2 (Fig. 1) - *Variations of branch volume and volume flow* for BrU1 and BrU2 respectively (Fig. 2). Due to the independent design of BrU1 and BrU2, both symmetrical and asymmetrical model geometry could be realized - *Discretization of the model geometry* to divide the model geometry into compartments
Balance of the molar fluxes	- Balancing the inflowing and outflowing molar fluxes for both convection and diffusion for each compartment - Diffusion process based on Fick’s first law
Specification of the initial and boundary conditions	- Initial condition: chamber filled with tracer gas - Boundary condition: a semipermeable membrane at the model input prevents tracer gas from flowing out
Simulation of the temporal concentration curves	- Model study was divided into three parts: - Part 1: Investigation of the influence of symmetrical volume change and flow rate on gas transport - Part 2: Investigation of the influence of asymmetrical volume change and flow rate on gas transport - Part 3: Investigation of the influence of changes in the diffusion coefficient under asymmetrical conditions
Analysis of the simulation data	- The simulated curves were adapted using monoexponential growth functions and the steady state value and growth constant were determined

### Geometrical assumptions

In previous studies, the anatomical data of the following authors were used for the dimensioning of the acinus:

- Haefeli-Bleuer and Weibel [30] (applied by Dutrieue et al. [25])
- Weibel et al. [31] (applied by Henry et al. [24])
- Majumdar et al. [32] and Florens et al. [33] (applied by Hasler et al. [28])
- Weibel [34] and Hansen and Ampaya [35] (applied by Paiva and Engel [26])

This work is based on the anatomical lung model of Weibel [34]. Here, the lungs are considered to be a symmetrically branched network of tubes or bronchi, comprising 23 generations. Each bronchus or bronchiole itself is divided into two daughter branches. The focus of the lung model was on gas transport processes within the acinus (generations 17 to 23) of the lung, corresponding to the end segments including the alveoli. These morphometric data could be applied directly to the method proposed in this paper to develop a numerical model consisting of two lung units connected in parallel, each of which could be regarded as dichotomous branching networks of the respiratory tract. The data determined by Weibel for generations 17 to 23 are marked with an asterisk in Table 2, alongside the data calculated from this for the model of this work.

**Table 2:** Important parameters of Weibel’s anatomical lung model [34] (marked with an asterisk) and the flow velocity calculated for each generation based on a volume flow in the trachea of *Q*_T_ = 250 ml/s. Parameters averaged over generations 17 to 23 are indexed with an “*M*.” The mean volume flow *Q*_M_ results from the mean diameter *d*_M_ and the mean flow velocity *u*_M_ as follows: $Q_{\mathrm M}=\frac\pi4\cdot d_{\mathrm M}^2\cdot u_{\mathrm M}$

Type of airway	Generation	Number of bronchioles (Br) per generation	Number of alveoli (Alv) per generation	Diameter of bronchioles		Length of bronchioles		Flow velocity and volume flow		
	z*	*m*_Br_(*z*)*	*m*_Alv_(*z*)*	*d*(*z*)*	*d*_M_	*l*(z)*	*l*_M_	*u*(*z*)	*u*_M_	*Q*_M_
				(cm)	(cm)	(cm)	(cm)	(cm/s)	(cm/s)	(cm^3^/s)
Respiratory bronchiole*	17	131,072	5	0.0543	0.0464	0.14	0.089	0.80	0.27	4.6 × 10^−4^
	18	262,144	8	0.0504		0.12		0.50		
	19	524,288	12	0.0474		0.10		0.30		
Alveolar duct*	20	1,048,576	20	0.0451		0.08		0.10		
	21	2,097,152	20	0.0434		0.07		0.10		
	22	4,194,304	20	0.0424		0.06		0.04		
Alveolar sacs*	23	8,388,608	17	0.0419		0.05		0.02		

Resting breathing conditions were simulated assuming a constant volume flow of *Q*_T_ = 250 ml/s (tidal volume 500 ml; breathing frequency 15 1/min) in the trachea. The velocity (*u*(*z*)) in a generation (*z*) was calculated by means of the following equation, assuming symmetrical branching (Table 2):

1 $$u\left( z \right) = \frac{{Q_{{\text{T}}} }}{{2^{z} \cdot A\left( z \right)}} = \frac{{Q_{{\text{T}}} }}{{2^{z} \cdot \frac{\pi }{4} \cdot d^{2} \left( z \right)}}$$

### Creation of a simple bronchial network

#### Simplification of Weibel’s model

**Fig. 1:**
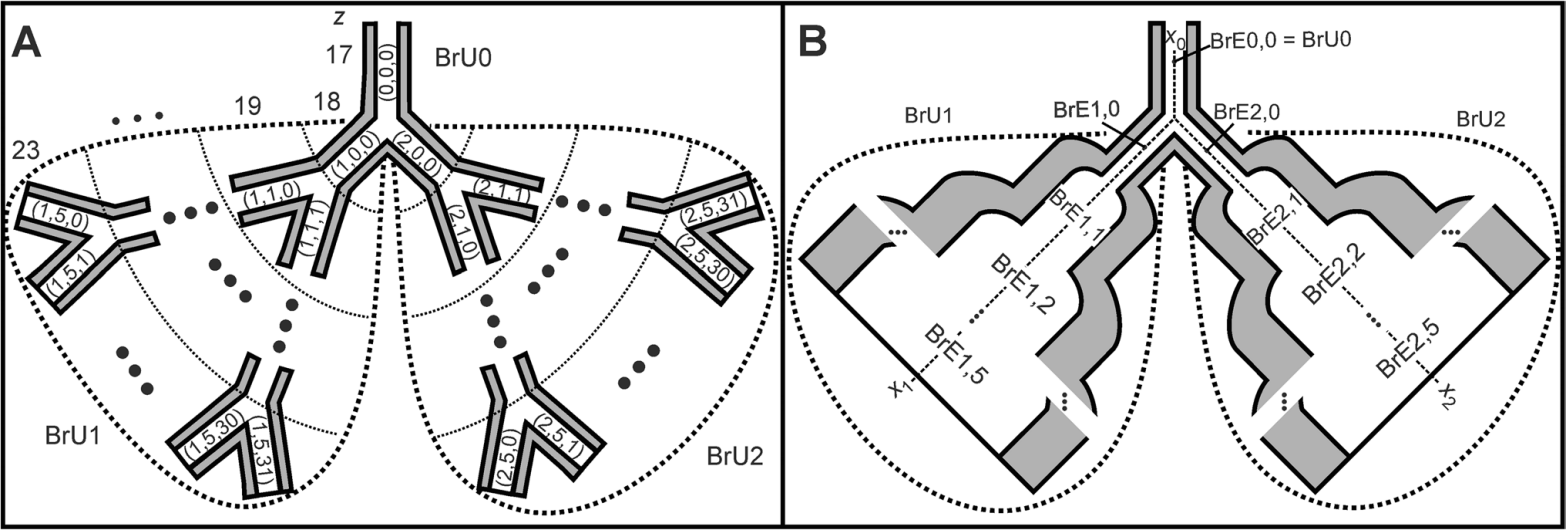
**a** This figure shows a simplified, symmetrically branched network of airways, beginning with one initial airway from generation 17 dividing into 64 bronchioles in generation 23, based on Weibel’s anatomical lung model [34]. This network is divided into three so-called branch units BrU0, BrU1, and BrU2. Three indices describe the position of each airway within this network. The first index is the branch unit, the second is the relative generation within this unit, and the third stands for the airway number within the respective, relative generation. Since BrU1 and BrU2 have a symmetrical structure, each comprised 6 generations. The walls of the airways are surrounded by alveoli (grey areas). **b** The symmetrically branched structure within each branch unit was merged to form the so-called branch elements BrE*i*,*j*. The index *i* stands for the branch unit; *j* stands for the respective generation within this unit

From the lung model derived by Weibel [34], the diameters, lengths, and flow velocities were first averaged over the selected generations 17 to 23. Subsequently, a dichotomous branched bronchial network starting from generation 17 was developed, in which all airways have averaged length (*l*_M_) and diameter (*d*_M_) (Fig. 1). Within the model, two branch units (BrU1 and BrU2) were defined, each comprising the generation 18 to 23. Thus, both branch units are supplied by the same mother branch unit (BrU0), which represents generation 17.

Within each branch unit BrU1 and BrU2, the symmetric dichotomous branching structure of each generation was merged to branch elements (BrE*i*,*j*) (Fig. 1), where the indices *i* and *j* indicate the branch unit (1,2) and the respective generation within the unit (from 0 to 5), respectively. All BrE*i*,*j* were modelled with cylindrical geometry and a smooth transition at the interface between adjacent generations. Each BrE*i*,*j*, which contains 2^*j*^ airways, was modelled with a single path. The cross-section of this pathway corresponds to the sum of the cross-sections of all parallel airways belonging to the generation, as shown in Fig. 1. The branch unit BrU0 consists of only one branch element.

A volume surrounding the branch elements (Fig. 1, grey area) modelled the alveoli. This volume varies with the gas flowing into the branch element via convection. The number of alveoli located in a single airway in the generations 17 to 23 is shown in Table 2. The mean number of alveoli (${\stackrel{\mathrm{-}}{\text{m}}}_{\mathrm{Alv}}$) per respiratory tract element was calculated over the number of alveoli per bronchiole (*m*_Alv_(*z*)) and the number of bronchioles (*m*_Br_(*z*)) in a generation *z* by using the following equation:

2 $${\overset-m}_\text{Alv}=\frac{\sum_{i=17}^{23}m_\text{Alv}\left(i\right)\cdot m_\text{Br}\mathrm{(}i\text{)}}{\sum_{i=17}^{23}m_\text{Br}\mathrm{(}i\mathrm{)}}$$

With the data from Table 2, ${\stackrel{\mathrm{-}}{\text{m}}}_{\text{Alv}}$ is 18.

Furthermore, the volume change over time resulting from gas flow was assumed to be equally distributed in all alveoli. With *Q*_T_ = 250 ml/s and a total number of alveoli in Weibel's model from 300 × 10^6^, each alveolus performs a volume change corresponding to the volume flow (*Q*_Alv_) of 8.3 × 10^−7^ cm^3^/s.

#### Variations of branch volume and volume flow

**Fig. 2:**
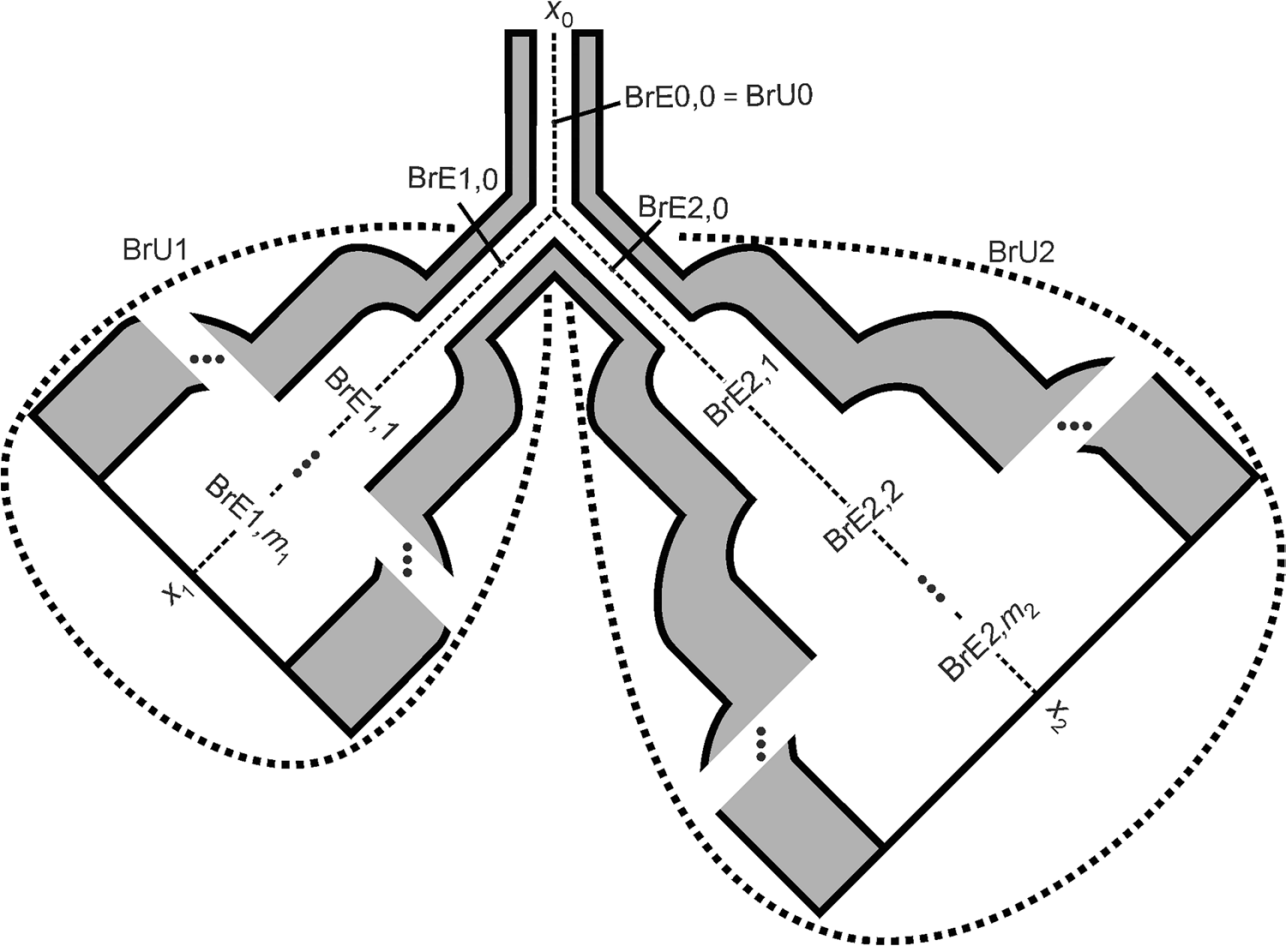
Asymmetric lung model consisting of three branch units BrU0, BrU1, and BrU2. The number of branch elements (*m*_1_ + 1) and (*m*_2_ + 1) within BrU1 and BrU2 depends on the volume flow at the inputs of these units

For the modelling of respiratory heterogeneity, both a symmetrical and an asymmetrical distribution of the bronchial tracts were considered, leading to different volumes for BrU1 and BrU2 (Fig. 2). However, regardless of the length and number of a BrE within a BrU, the above average number of alveoli per respiratory pathway and the alveolar volume flow were maintained unchanged.

Thus, the total volume flow (*Q*_BrU*i*_) at the input of the branch unit is proportional to the number of branch elements within a BrU. In the model presented in this study, in contrast to the simplified Weibel model, a BrU consists of *s*_BrU*i*_ + 1 (*s*_BrU*i*_ ≥ 0) BrEs of length *l*_M_ and one BrE of length *a*_BrU*i*_ × *l*_M_ (0 ≤ *a*_BrU*i*_ ≤ 1). *Q*_BrU*i*_ can be derived from the combined volume flows *Q*_Alv_ of all alveoli and can be calculated with Eq. 3.

3 $$Q_{\text{BrU}i}\text{=}\left(\sum_{i=\text{0}}^{s_{\text{BrU}i}}2^i\text{+}a_{\text{BrU}i}\cdot2^{s_{\text{BrU}i}+\text{1}}\right)\cdot{\overset-m}_\text{Alv}\cdot Q_\text{Alv}$$

If *a*_BrU*i*_ is replaced by 2^*b*^ − 1 (0 ≤ *b* ≤ 1), Eq. 3 can be simplified as follows:

4 $$Q_{\text{BrU}i}\mathrm{=}\left(2^{s_{\text{BrU}i}\mathrm{+}\text{b}+\mathrm{1}}\text{-1}\right)\cdot{\overset-m}_\text{Alv}\cdot Q_\text{Alv}$$

By means of Eq. 4, the term ${\stackrel{\mathrm{\sim }}{\text{s}}}_{{\mathrm{BrU}}i}$ = *s*_BrU*i*_ + *b* can be determined with:

5 $${\widetilde s}_{\text{BrU}i}\mathrm{=}{\text{log}}_2\left[\text{0.5}\cdot\left(\frac{Q_{\text{BrU}i}\mathrm{/}Q_\text{Alv}}{{\overset-m}_\text{Alv}}+\mathrm{1}\right)\right]$$

The parameters *s*_BrU*i*_ and *a*_BrU*i*_ can now be determined from ${\stackrel{\mathrm{\sim }}{\text{s}}}_{{\mathrm{BrU}}i}$ as follows:

6 $$s_{{{\text{BrU}}i}} = \left\lfloor {\tilde{s}_{{{\text{BrU}}i}} } \right\rfloor$$

7 $$a_{\text{BrU}i}\text{=}2^{{\widetilde s}_{\text{BrU}i}-s_{\text{BrU}i}}\mathrm{ - 1}$$

The total length (*l*_BrU*i*_) of a BrU*i* is thus:

8 $$l_{\mathrm{BrU}i}=\left(s_{\mathrm{BrU}i}+a_{{}_{\mathrm{BrU}i}}+1\right)\cdot l_{\mathrm M}$$

Since the cross-sectional shape of a branch unit is predetermined, this total length is dependent on the volume of the branch unit and corresponds to the diffusion path length of the gas within this branch unit.

The alveoli are evenly distributed on the surface of the airways of a BrE (Fig. 1). Accordingly, the total number of alveoli in each branch unit (*m*_Alv,BrU*i*_) is:

9 $$m_{\text{Alv,BrU}i}\text{=}\left(2^{{\widetilde s}_{\text{BrU}i}+\text{1}}\text{ - 1}\right)\cdot{\overset-m}_\text{Alv}$$

The volume of a BrU*i* (*V*_BrU*i*_(*t*)) according to Eq. 10 is the sum of the time-constant volumes of all airways and the time-dependent volumes of all alveoli within the BrU*i*.

10 $$V_{\text{BrU}i}\left(t\right)=\left(2^{{\widetilde s}_{\text{BrU}i}+\text{1}}-1\right)\cdot\lbrack\frac\pi4\cdot d_\text{M}^2\cdot l_\text{M}\mathrm{+}{\overset-m}_\text{Alv}\cdot\left(V_\text{Alv}\text{+}Q_\mathrm{Alv}\cdot\text{t}\right)\rbrack$$

The base volume of an alveolus (*V*_Alv_) corresponds to that of a sphere. Its diameter (*d*_Alv_) can be determined with the equation *d*_Alv_ = 1.54 × 10^−3^∙(3 l)^1/3^ from Weibel [34]. Here, it was assumed that the resting volume of the lungs is about 3 l [36].

Equation 10 shows that the ratios of BrU volumes correspond to the ratios of their number of airways:

11 $$f\text{ = }\frac{{\text{V}}_\mathrm{BrU1}\text{(}\mathrm{t}\text{)}}{{\mathrm{V}}_\text{BrU2}\mathrm{(}\text{t}\mathrm{)}}=\frac{2^{{\widetilde{\text{s}}}_\mathrm{BrU1}+\text{1}}\mathrm{-1}}{2^{{\widetilde{\text{s}}}_\mathrm{BrU2}+\text{1}}\mathrm{-1}}$$

Furthermore, this equation shows that in the model presented, these ratios are independent of time. This makes it possible to display diagrams in relation to a volume ratio in order to compare these results (see chapter results).

Under symmetrical conditions where the volumes BrU1 and BrU2 are identical at all times, *f* is defined based on a reference value ${\stackrel{\mathrm{\sim }}{\text{s}}}_{\text{ref}}$ = 3.99 (and a corresponding reference volume *V*_ref_) where the total amount of alveoli results in a total volume flow (*Q*_M_) (Table 2) of:

12 $$Q_\text{M}\text{ =}u_\text{M}\cdot\pi\text{/4}\cdot d_\text{M}^2$$

Thus, in such cases:

13 $$f_\text{sym}=\text{ }\frac{2^{{\widetilde{\text{s}}}_\text{BrU1,2}+\text{1}}\text{-1}}{2^{{\widetilde{\text{s}}}_\text{ref}+\text{1}}\mathrm{-1}}\frac{}{}$$

#### Discretization of the model geometry

**Fig. 3:**
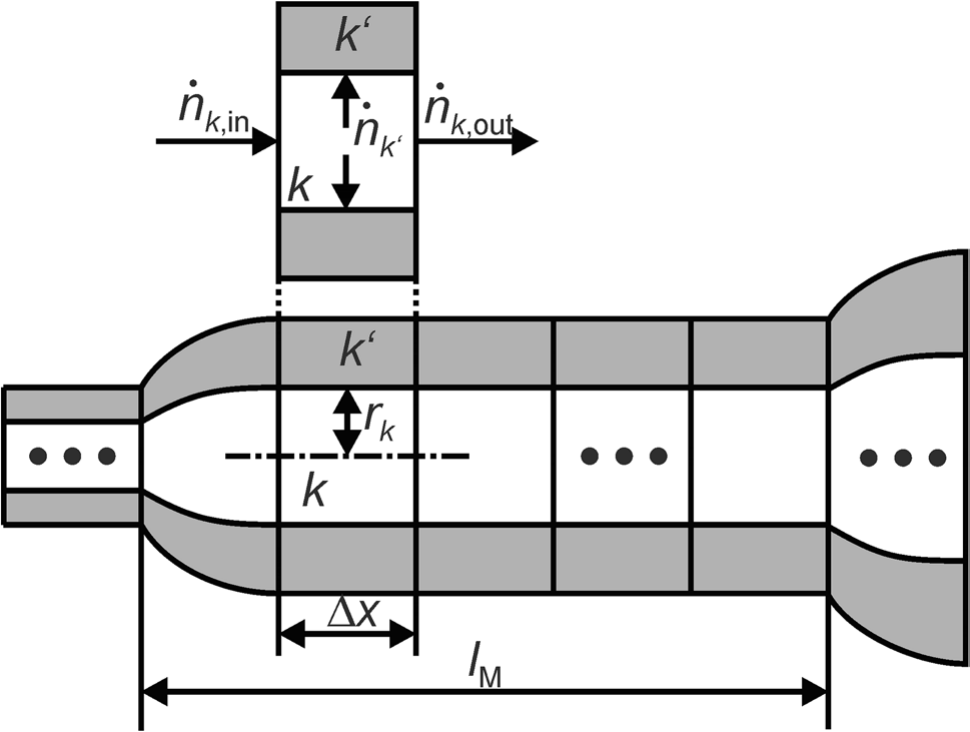
Molar fluxes ${\dot{\text{n}}}_{{k}\text{,in}}$ and ${\dot{\text{n}}}_{k\text{,out}}$ flowing into and out of the compartment *k* of a locally discretized BrE of length *l*_M_ and the flux $\dot{n}_{k\prime }$ flowing into the surrounding alveoli *k*′

The model described above was discretized along the respective tube axes *x*_0_, *x*_1_, and *x*_2_ of the BrUs (Fig. 2). The local resolution (Δ*x*) is 10 μm, which allows a high spatial resolution and a reasonable simulation time. As a result of local discretization, both the BrEs and the alveoli surrounding their surfaces are divided into compartments. A single BrE with a length of *l*_M_ = 0.089 cm, shown in Fig. 3, thus consists of 89 compartments and as many surrounding alveolar compartments. The diameters at the transitions of these BrEs were adjusted to obtain a linear and non-erratic cross-sectional shape. This correction is also applied to the branching point, where the mother branch unit BrU0 is divided into the two branch units BrU1 and BrU2.

### Balance of the molar fluxes

In this work, Taylor dispersion, gas exchange, and adsorption and absorption processes on the mucous membranes were not considered. As a result, the gas transport only depended on convection and molecular diffusion. The numerical model, based on the finite difference method, was implemented in MATLAB® R2019b (MathWorks®, Natick). Each compartment (*k*) along *x*_*i*_ of a BrU*i* was assigned a corresponding alveolar compartment (*k’*). For each *k* as well as *k’*, the inflowing and outflowing molar fluxes were balanced. Therefore, the time-dependent changes in the molar quantity $(\frac{{{\text{d}}n_{k} (t)}}{{{\text{d}}t}}{\text{or}}\frac{{{\text{d}}n_{k\prime } (t)}}{{{\text{d}}t}})$ of a tracer gas in *k* or *k’* can be described mathematically as:

14 $$\frac{{{\text{d}}n_{k} (t)}}{{{\text{d}}t}} = \dot{n}_{{k,{\text{in}}}} (t) - \dot{n}_{{k,{\text{out}}}} (t) - \dot{n}_{k\prime } (t)$$

15 $$\frac{{{\text{d}}n_{k\prime } (t)}}{{{\text{d}}t}} = \dot{n}_{k\prime } (t)$$

Parameters $\dot{n}_{{k,{\text{in}}}} (t)$ and $\dot{n}_{{k,{\text{out}}}} (t)$ are the inflowing and outflowing molar fluxes of the adjacent branch element compartments, whereas $\dot{n}_{k\prime } (t)$ is the molar flux between *k* and *k’*. These three molar fluxes can be described in detail as follows:

16 $$\dot{n}_{{k{\text{,in}}}} (t) = Q_{{k{\text{,in}}}} \cdot c_{k - 1} (t) - D \cdot A_{{k{\text{,in}}}} \cdot \frac{{c_{k} (t) - c_{k - 1} (t)}}{\Delta x}$$

17 $$\dot{n}_{{k{\text{,out}}}} (t) = Q_{{k{\text{,out}}}} \cdot c_{k} (t) - D \cdot A_{{k{\text{,out}}}} \cdot \frac{{c_{k + 1} (t) - c_{k} (t)}}{\Delta x}$$

18 $$\dot{n}_{k\prime } (t) = Q_{k\prime } \cdot c_{k} (t) - D \cdot A_{k\prime } \cdot \frac{{c_{k\prime } (t) - c_{k} (t)}}{{r_{k} }}$$

In general, each of these molar fluxes consists of a convection part, which depends on the volume flow and the concentration (*c*), and a diffusion part, which depends on the molecular diffusion coefficient, the flow area (*A*), and the discretized concentration gradient between adjacent compartments. The diffusion equation is based on Fick’s first diffusion law. *Q*_*k’*_ is the volume flow into the alveoli; *A*_*k’*_ is the surrounding surface of *k*.

The diffusion path length of a compartment in the axial direction is defined by Δ*x* while for the diffusion path between *k* and *k'*, the radius of the compartment (*r*_*k*_) was taken for the sake of simplicity (Fig. 3). Since *D* is only defined for mixtures of two gas components, the gas mixture used in this work consists of just one tracer gas and N_2_ as the carrier component. The minimum and maximum diffusion coefficients for this gas mixture are between 0.1 cm^2^/s if SF_6_ is used and 0.6 cm^2^/s for He [26]. For the simulations with different BrU volumes, a test tracer gas with a diffusion coefficient of 0.3 cm^2^/s was chosen. At the branching point, two molar fluxes leave BrU0, one into BrU1 and the other into BrU2.

The molar quantity *n*_*k*_ in Eq. 14 was replaced by its volume (*V*_*k*_), and concentration (*c*_*k*_) of $n_k\left(t\right)=V_k\cdot c_k(t)$. *V*_*k*_ was considered constant over time. Furthermore, *c*(*t*) can be rewritten with the time-dependent mole fractions (*χ*(*t*)) and the molar volume (V_m_) to $c\left(t\right)=\frac{\upchi (t)}{{\mathrm{V}}_{\mathrm{m}}}$. Consequently, the differential Eq. (14) can also be described as follows:

19 $$\frac{{{\text{d}}\chi_{k} \left( t \right)}}{{{\text{d}}t}} = \frac{1}{{V_{k} }} \cdot \left[ {Q_{{k,{\text{in}}}} \cdot \chi_{k - 1} \left( t \right) - Q_{{k,{\text{out}}}} \cdot \chi_{k} \left( t \right) - Q_{k\prime } \cdot \chi_{k} \left( t \right) + D \cdot \left( { - \frac{{\chi_{k} \left( t \right) - \chi_{k - 1} \left( t \right)}}{\Delta x} \cdot A_{{k,{\text{in}}}} + \frac{{\chi_{k + 1} \left( t \right) - \chi_{k} \left( t \right)}}{\Delta x} \cdot A_{{k,{\text{out}}}} + \frac{{\chi_{k\prime } \left( t \right) - \chi_{k} \left( t \right)}}{{r_{k} }}A_{k\prime } } \right)} \right]$$

Since the volume of the alveolar compartments (*V*_*k’*_) depends on *Q*_*k’*_, which corresponds to the volume change $\frac{{{\text{d}}V_{k\prime } (t)}}{{{\text{d}}t}}$, the molar fraction in the alveolar compartments can be described by:

20 $$\frac{\mathrm{d}{\upchi }_{{k}^{^{\prime}}}(t)}{\mathrm{d}t}=\frac{1}{{V}_{{k}^{^{\prime}}}(t)}\cdot ({Q}_{{k}^{^{\prime}}}\cdot {\upchi }_{k}\left(t\right)-D\cdot {A}_{{k}^{^{\prime}}}\cdot \frac{{\upchi }_{{k}^{^{\prime}}}\left(t\right)-{\upchi }_{k}\left(t\right)}{{r}_{k}}-\frac{\mathrm{d}{V}_{{k}^{^{\prime}}}\left(t\right)}{\mathrm{d}t}\cdot {\upchi }_{{k}^{^{\prime}}}(t))$$

In consideration of all *k* and *k*’, this leads to a differential equation system. The numerical model was solved with the ode45 function.

For the subsequent evaluation of the simulation results, the tracer gas volumes in the BrU*i*s were calculated from the simulated mole fractions:

21 $$V_{{g,{\text{BrU}}i}} (t) = \sum\limits_{\forall k} {\chi_{{{\text{BrU}}i,k}} (t) \cdot V_{{{\text{BrU}}i,k}} + \sum\limits_{\forall k\prime } {\chi_{{{\text{BrU}}i,k\prime }} (t) \cdot } } V_{{{\text{BrU}}i,k\prime }} (t)$$

### Specification of the initial and boundary conditions

**Fig. 4:**
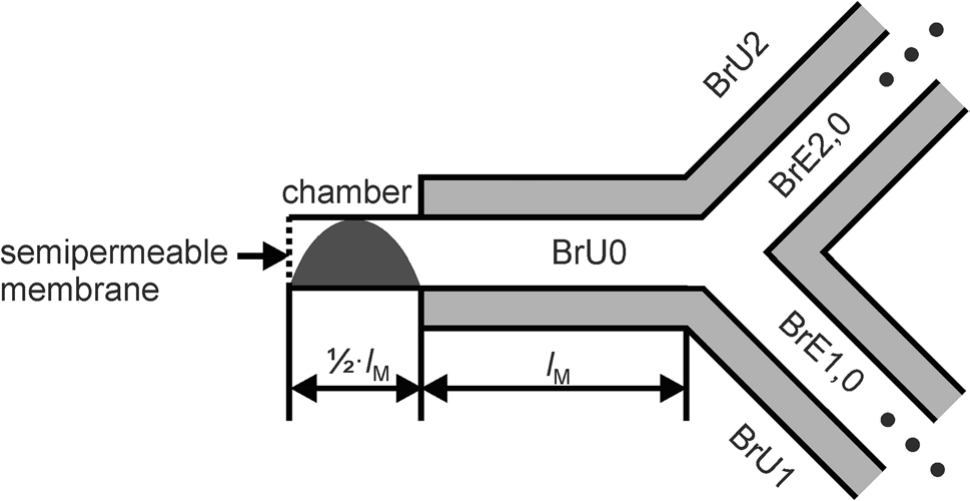
The branch unit BrU0 is extended by a chamber that has no alveoli and a length of 1/2∙*l*_M_. In this chamber, at time *t* = 0 s, there is the tracer gas whose spatial distribution corresponds to a sinusoidal half-wave (marked in black). There is no tracer gas in the rest of the model at this time. At the entrance, a semipermeable membrane (dotted line) was modelled so that N_2_ could flow into the model but the tracer gas could not flow out

For the implementation of the initial and boundary conditions, the length of the branch unit BrU0 was extended by *l*_M_/2 (Fig. 4). This additional chamber contained the complete tracer gas at the simulation time *t* = 0 s, while the rest of the model was filled with the carrier gas N_2_. At this time, the tracer gas was distributed over the length of the chamber in the shape of a sinusoidal half-wave, as shown in Fig. 4, as it had proved to be resistant to numerical smearing when convection was also taken into account. Due to the length of the chamber and the shape of the tracer gas distribution function, the total amount of the tracer (*V*_g,Tr_) is 4.78 × 10^−5^ ml.

To prevent the tracer gas from leaving the model, a semi-permeable membrane was implemented at the input of the model. Thus, only the carrier gas N_2_ was able to pass through this membrane.

### Simulation of the temporal concentration courses

The aim of this work was to investigate the effect of geometry, flow rate and diffusion properties on gas transport. Both symmetrical and asymmetrical geometry were considered separately. To investigate the effect of flow rate, simulations were performed, in which pure diffusion and additional convection under identical geometrical conditions were taken into account. Under these considerations, this study was divided into three parts:

1. Investigation of the influence of symmetrical volume change and flow rate on gas transport.
2. Investigation of the influence of asymmetrical volume change and flow rate on gas transport.
3. Investigation of the influence of changes in the diffusion coefficient under asymmetrical conditions.

### Part 1 – Investigation of the influence of symmetrical volume change and flow rate on gas transport

For the first six simulations, the volume of BrU0 including the chamber was kept constant at 3.31 × 10^−4^ ml and a symmetrical model (*V*_BrU1_ = *V*_BrU2_ = *V*_BrU1,2_) without convection, but pure diffusion was assumed. Using pure diffusion means that the volumes of all compartments do not change over time. The time-dependent concentration curves of the test tracer gas were simulated with the six different volumes *V*_BrU1,2_ given in Table 3. The molecular diffusion coefficient was set to 0.3 cm^2^/s, the simulation time to 30 s.

**Table 3:** Parameters used in Parts 1 to 3 of the study. Simulations 1 to 6 were each performed for pure diffusion (*V*_BrU1_ and *V*_BrU2_ are constant over time) and additional convection (*V*_BrU1_ and *V*_BrU2_ correspond to the values at *t* = 0 s). *f* is the volume ratio between *V*_BrU1_ and *V*_BrU2_. *f*_sym_ is the ratio of *V*_BrU1_ to the reference volume *V*_ref_ of 7.76 × 10^−3^ ml. For more information, see text

sim	Part 1					Part 2					Part 3				
	*f*_sym_	*V*_BrU1_ *V*_BrU2_	Q_BrU1_ Q_BrU2_	u_BrU1_ u_BrU2_	*D*	*f*	*V*_BrU1_ *V*_BrU2_	Q_BrU1_ Q_BrU2_	u_BrU1_ u_BrU2_	*D*	*f*	*V*_BrU1_ *V*_BrU2_	Q_BrU1_ Q_BrU2_	u_BrU1_ u_BrU2_	*D*
		(ml)	(ml/s)	(cm/s)	(cm^2^/s)		(ml)	(ml/s)	(cm/s)	(cm^2^/s)		(ml)	(ml/s)	(cm/s)	(cm^2^/s)
1	0.074	$5.76\times {10}^{-4}$ $5.76\times {10}^{-4}$	$\text{3.41 }\times {10}^{-5}$ $\text{3.41 }\times {10}^{-5}$	0.02 0.02	0.1 0.3 0.6	0.074	$\text{5.76 }\times {10}^{-4}$ $\text{7.76 }\times {10}^{-3}$	$\text{3.41 }\times {10}^{-5}$ $\text{4.60 }\times {10}^{-4}$	0.02 0.27	0.1 0.3 0.6	0.5	3.88 $\times$ 10^−3^ 7.76 $\times$ 10^−3^	2.3 $\times$ 10^−4^ 4.6 $\times$ 10^−4^	0.135 0.27	0.100
2	0.796	$6.19\times {10}^{-3}$ $\text{6.19}\times {10}^{-3}$	$\text{3.67 }\times {10}^{-4}$ $\text{3.67 }\times {10}^{-4}$	0.215 0.215		0.796	$\text{6.19 }\times \, {10}^{-3}$ $\text{7.76 }\times {10}^{-3}$	$\text{3.67 }\times {10}^{-4}$ $\text{4.60 }\times {10}^{-4}$	0.215 0.27						0.225
3	1.000	$\text{7.76}\times {10}^{-3}$ $\text{7.76 }\times \, {10}^{-3}$	$\text{4.60 }\times {10}^{-4}$ $\text{4.60 }\times {10}^{-4}$	0.27 0.27		1.000	$\text{7.76 }\times {10}^{-3}$ $\text{7.76 }\times {10}^{-3}$	$\text{4.60 }\times {10}^{-4}$ $\text{4.60 }\times {10}^{-4}$	0.27 0.27						0.350
4	1.519	$\text{1.18 }\times \, {10}^{-2}$ $\text{1.18 }\times \, {10}^{-2}$	$\text{6.99 }\times {10}^{-4}$ $\text{6.99 }\times {10}^{-4}$	0.41 0.41		1.519	$\text{1.18 }\times {10}^{-2}$ $\text{7.76 }\times {10}^{-3}$	$\text{6.99 }\times {10}^{-4}$ $\text{4.60 }\times {10}^{-4}$	0.41 0.27						0.475
5	2.241	$\text{1.74 }\times \, {10}^{-2}$ $\text{1.74 }\times \, {10}^{-2}$	$\text{1.03 }\times \, {10}^{-3}$ $\text{1.03 }\times {10}^{-3}$	0.605 0.605		2.241	$\text{1.74 }\times {10}^{-2}$ $\text{7.76 }\times {10}^{-3}$	$\text{1.03 }\times {10}^{-3}$ $\text{4.60 }\times {10}^{-4}$	0.605 0.27						0.600
6	2.963	$\text{2.30 }\times \, {10}^{-2}$ $\text{2.30 }\times \, {10}^{-2}$	$\text{1.36 }\times {10}^{-3}$ $\text{1.36 }\times {10}^{-3}$	0.8 0.8		2.963	$\text{2.30 }\times {10}^{-2}$ $\text{7.76 }\times {10}^{-3}$	$\text{1.36 }\times {10}^{-3}$ $\text{4.60 }\times {10}^{-4}$	0.8 0.27						

The next simulations were performed under the same conditions as described above, but this time considering convection. The total volume flows (*Q*_BrU1_ and *Q*_BrU2_) and the corresponding flow velocities (*u*_BrU1_ and *u*_BrU2_) at the input of the branch units BrU1 and BrU2 are listed in Table 3. The range of flow velocities corresponds to the different conditions of generations 17 to 23 (Table 2). In this case, the volumes of all branch units changed over time. Therefore, the volumes given in Table 3 refer to the beginning of the simulation (*t* = 0 s). The diffusion coefficient was initially set to 0.3 cm^2^/s in order to compare the results with those for pure diffusion. Additional simulations with diffusion coefficients of 0.1 cm^2^/s and 0.6 cm^2^/s were carried out to analyze the interactions between the diffusion coefficient and volume flow in the context of a sensitivity analysis.

### Part 2 – Investigation of the influence of asymmetrical volume change and flow rate on gas transport

Asymmetrical changes in volume were realized by varying the volume of one branch unit (BrU1), while the other branch unit (BrU2) was kept constant. The range of volume changes is listed in Table 3. The volumes of BrU0 and BrU2 were fixed to 3.31 × 10^−4^ ml and 7.76 × 10^−3^ ml, respectively. The time-dependent concentration profiles were first simulated with pure diffusion, then with both diffusion and convection. The volume flows at the input of BrU1 corresponded to those in Table 3 for each simulation, while the volume flow of BrU2 was set constant to *Q*_M_ = 4.60 × 10^−4^ ml/s (Table 2). As in Part 1 of the study, the diffusion coefficient of 0.3 cm^2^/s was used for pure diffusion and additional convection in order to compare the results. As in Part 1, additional simulations were carried out with the diffusion coefficients 0.1 cm^2^/s and 0.6 cm^2^/s with diffusion and convection.

### Part 3 – Investigation of the influence of changes in the diffusion coefficient under asymmetrical conditions

This part of the study examined the influence of the diffusion coefficient. Again, in the first five simulations, only diffusion without convection was considered, while in the following five simulations, convection was added. The volumes of BrU0 including the chamber, BrU1, and BrU2 were set to 3.31 × 10^−4^ ml, 3.88 × 10^−3^ ml, and 7.76 × 10^−3^ ml, respectively. For each of the five simulations with and without convection, the diffusion coefficients given in Table 3 were used. For the simulation with convection, the mean volume flow of *Q*_M_ = 4.6 × 10^−4^ ml/s was used at the input of BrU2 (Table 2); a volume flow of 2.3 × 10^−4^ ml/s was used for BrU1, corresponding to half of BrU2.

### Analysis of the simulation data

**Fig. 5:**
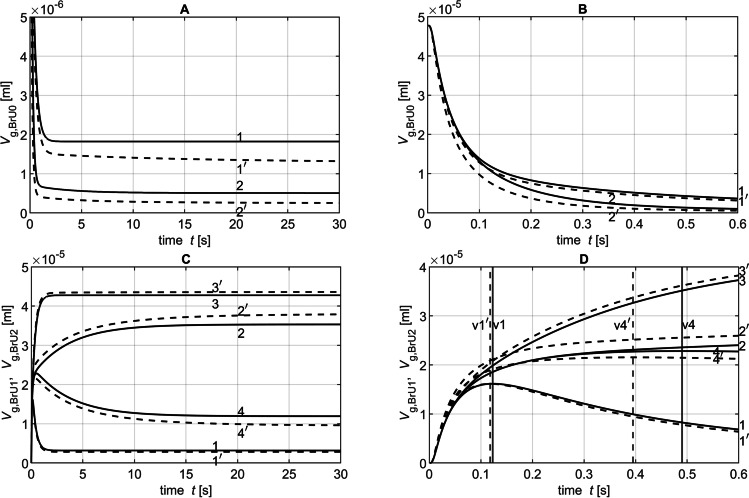
Time diagrams of *V*_g,BrU0_(*t*) (**A**, **B**), *V*_g,BrU1_(*t*) (**C**, **D** curves 1, 1′, 2 and 2′) and *V*_g,BrU2_(*t*) (**C**, **D** curves 3, 3′, 4 and 4′) from Part 2 of the parameter study with (dotted lines) and without (solid lines) convection for different time intervals. The curves 1, 1′, 3, and 3′ correspond to the conditions of simulation 1 in Table 3; the curves 2, 2′, 4, and 4′ correspond to those of simulation 6. The vertical lines v1 and v1′ mark the transition from phase 1 to phase 2 of the corresponding curves 1 and 1′. Accordingly, the vertical lines v4 and v4′ mark the phase transitions of the curves 4 and 4′

First, *V*_g,BrU*i*_(*t*) in the units BrU0, BrU1, and BrU2 were calculated from the time-dependent concentration curves (Eq. 21). Then, in the case of asymmetric conditions, *V*_g,BrU*1*_(*t*) and *V*_g,BrU*2*_(*t*) were divided into two phases, as shown in the results (Fig. 5).

In general, gas flows from BrU0 into the units BrU1 and BrU2 over the entire time interval 0 s ≤ *t* ≤ 30 s used for the simulations. This large time interval was chosen to allow *V*_g,BrU*i*_(*t*) to reach its steady-state value. When one BrU has a lower total volume than the other, then, in the first phase, both units have a net inflow of the tracer gas from BrU0. However, when the tracer gas concentration in the BrU with the lower total volume reaches its maximum value at time *t*_p1_, this net inflow into this unit stops and the second phase begins. As a result, the net inflow of tracer gas into the unit with the larger total volume now has two sources, one from BrU0 and the other from the unit with the smaller total volume. In this case, the time intervals for phase 1 and phase 2 were defined as 0 s ≤ *t* ≤ *t*_p1_ for phase 1 and *t*_p1_ ≤ *t* ≤ 30 s for phase 2.

For the sake of simplicity, *V*_g,BrU*i*_(*t*) is described with two exponential growth functions given in Eq. 22, each valid for only one phase but with different parameters for the start value *V*_g,BrU*i*_(*t*_start_), the end value *V*_g,BrU*i*_(*t*_end_), and the growth constant (*λ*_BrU*i*_). For phase 1 *t*_start_ = 0 s, *t*_end_ = *t*_p1_ and for phase 2 *t*_start_ = *t*_p1_, *t*_end_ = 30 s.

22 $$V_{\mathrm g,\mathrm{BrU}i}\left(t\right)=V_{\mathrm g,\mathrm{BrU}i}\left(t_{\mathrm{end}}\right)+\lbrack V_{\mathrm g,\mathrm{BrU}i}\left(t_{\mathrm{start}}\right)-V_{\mathrm g,\mathrm{BrU}i}\left(t_{\mathrm{end}}\right)\rbrack\cdot\mathrm e^{-\lambda_{\mathrm{BrU}i}\cdot t}$$

Each exponential growth function was determined from the simulated data set of the corresponding phase using the curve fitting tool provided in MATLAB R2019b. The Levenberg–Marquardt algorithm was chosen, which is the commonly used method for fitting nonlinear data sets. The parameters of the growth functions were only determined for BrU0 and BrU1.

## Results

### Part 1 — Investigation of the influence of symmetrical volume change and flow rate on gas transport

**Fig. 6:**
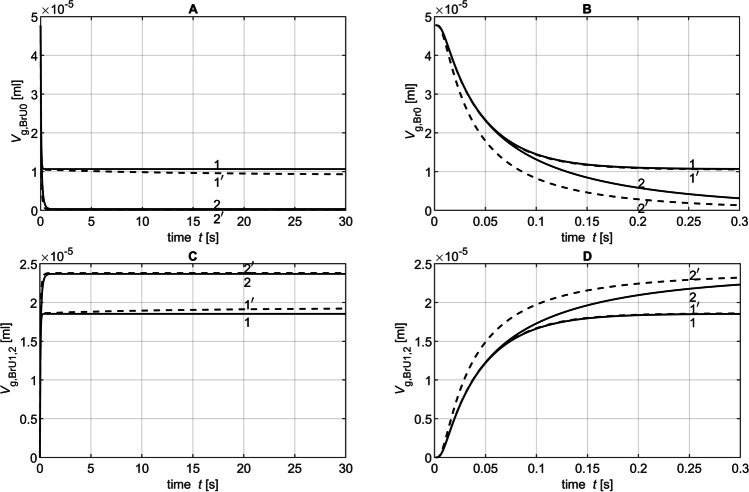
Time diagrams of *V*_g,BrU0_(*t*) (**A**, **B**) and *V*_g,BrU1,2_(*t*) (**C**, **D**) from the Part 1 of the parameter study for simulations 1 (curves 1 and 1′) and 6 (curves 2 and 2′) from Table 3 with (dotted lines) and without (solid lines) convection for different time intervals

**Fig. 7:**
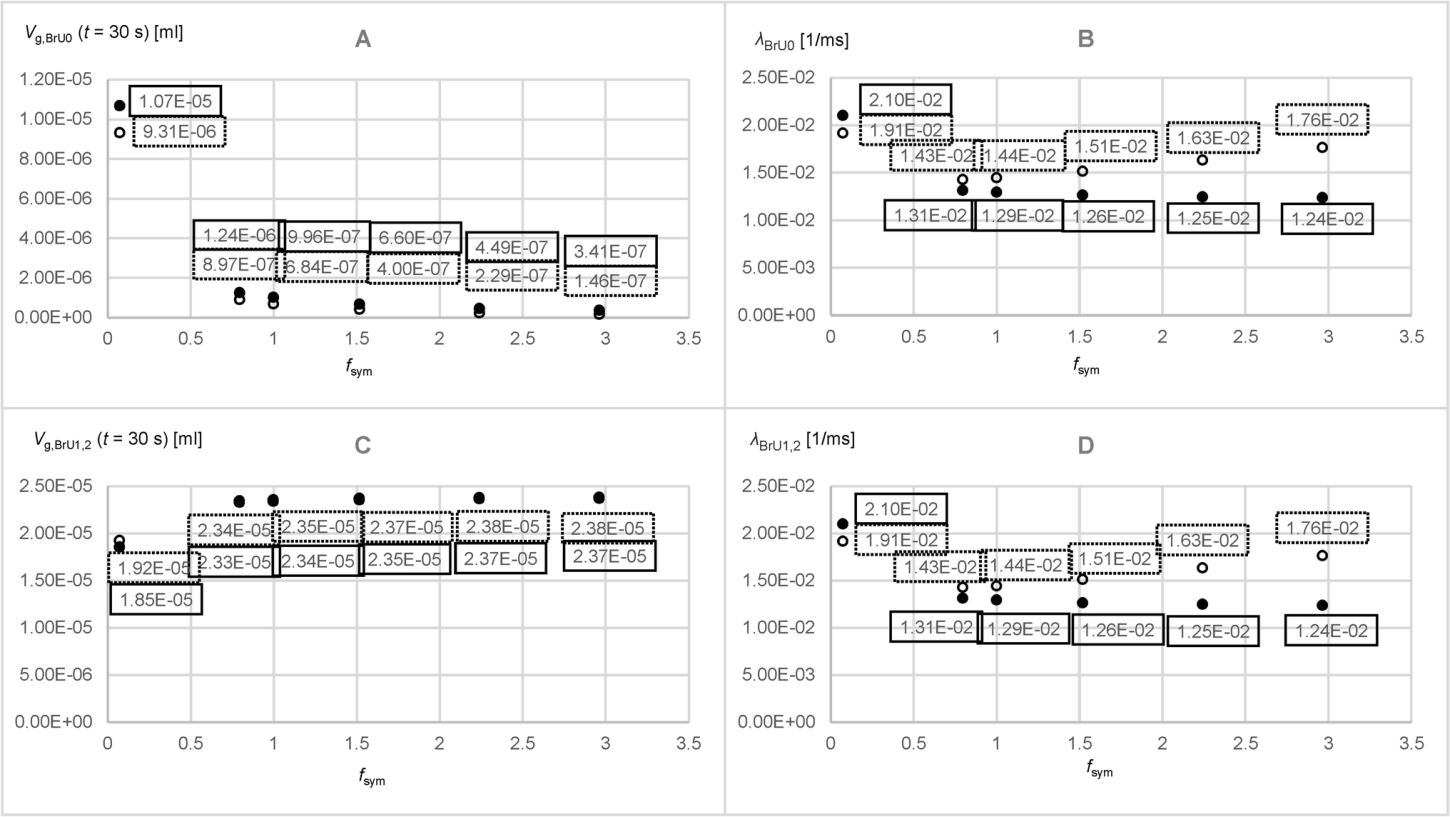
Parameters obtained from the time diagrams *V*_g,BrU0_(*t*) and *V*_g,BrU1_(*t*) of Part 1 of the parameter study according to Eq. 22 for pure diffusion (filled circles) and with additional convection (unfilled circles)

Figure 6 shows the time-dependent curves with and without the convection of the tracer gas volumes *V*_g,BrU0_(*t*) and *V*_g,BrU1_(*t*) simulated for the volumes *V*_BrU1_ = 5.76 × 10^−4^ ml and *V*_BrU1_ = 2.30 × 10^−2^ ml with a diffusion coefficient of 0.3 cm^2^/s. Since symmetrical conditions were assumed for BrU1 and BrU2, the tracer gas volumes *V*_g,BrU1_(*t*) and *V*_g,BrU2_(*t*) were also identical, so both volumes could be substituted with *V*_g,BrU1,2_(*t*). It could be observed that all curves start with a plateau, since the tracer gas needed some time to move from BrU0 to the branching point. At the end of the simulation after *t* = 30 s, the tracer gas volumes *V*_g,BrU*i*_(*t* = 30 s) for the branch units BrU0, BrU1, and BrU2 had already reached their steady state values (*V*_g,st,BrU*i*_) when only diffusion was responsible for the tracer gas transport. As expected, the distribution of the total tracer gas volume *V*_g,Tr_ = 4.78 × 10^−5^ ml within a branch unit at steady state depends on the ratio of its volume *V*_BrU*i*_ to the total volume of the model *V*_tot_ (Fig. 7A and C):

23 $$V_{\text{g,st,BrU}i}\text{=}\frac{V_{\text{BrU}i}}{V_\text{tot}}\cdot4.78\times10^{-5}\mathrm{ml}$$

When gas transport was realized also by convection, more tracer gas flows from BrU0 into the branch units BrU1,2, resulting in reduced values *V*_g,BrU*i*_(*t* = 30 s) at the end of the simulation time (Fig. 7A and C). Furthermore, at large volume flow, as shown in Fig. 6 (curves 2′), the branch units BrU1 and BrU2 were filled with tracer gas more quickly if convection was also involved in tracer gas transport. Figure 7B and D show that with the exception of very low volume flows, the larger the volume or volume flow, the larger the *λ* compared to those without convection. For small volume flows like *Q*_BrU1,2_ = 3.41 × 10^−5^ ml/s, *V*_g,BrU0_(*t*) and *V*_g,BrU1,2_(*t*) were almost identical to those for pure diffusion in the first 0.3 s (Fig. 6B, D). However, compared to pure diffusion at this volume of *V*_BrU1,2_ = 5.76 × 10^−4^ ml, with convection, tracer gas kept flowing from BrU0 into BrU1,2 even after 0.3 s (Fig. 6A, C). This led to a smaller *λ* than that found with pure diffusion (Fig. 7B, D). The results for the simulations with diffusion coefficients, 0.1 cm^2^/s and 0.6 cm^2^/s, were explored in Part 2.

Table 4 shows the mean values and standard deviations of the mean coefficients of determination ($\overline{{\text{R} }^{2}}$) and root-mean-square errors ($\overline{\text{RMSE}}$) from the curve fittings for the three parts of the analysis. They were averaged over all simulations for BrU0 and BrU1 and the respective phases with and without convection.

**Table 4:** Mean values and standard deviations of the coefficients of determination ($\overline{{\text{R} }^{2}}$) and root-mean-square errors ($\overline{\text{RMSE}}$) with the corresponding minimum and maximum values from the trend line determinations of *V*_g,BrUi_(*t*) for BrU0 and BrU1 of Parts 1 to 3 each without (first value) and with convection (second value). The diffusion coefficient was 0.3 cm^2^/s

Part	Branch unit	$\overline{{\text{R} }^{2}}$		$\overline{\text{RMSE}}$(ml)	
		Mean ± SD	Min|max	Mean ± SD	Min|max
1	BrU0	0.9903 ± 0.2016 × 10^−2^ 0.9577 ± 0.8025 × 10^−1^	0.9876|0.9925 0.7939|0.9907	1.6 × 10^−7^ ± 3.4 × 10^−8^ 2.2 × 10^–7^ ± 1.6 × 10^−7^	9.8 × 10^−8^|1.9 × 10^−7^ 1.4 × 10^−7^|5.4 × 10^−7^
	BrU1	0.9903 ± 0.2016 × 10^−2^ 0.9577 ± 0.8025 × 10^−1^	0.9876|0.9925 0.7939|0.9907	7.8 × 10^−8^ ± 1.7 × 10^−8^ 1.1 × 10^−7^ ± 8.0 × 10^−8^	4.9 × 10^−8^|9.5 × 10^8^ 7.1 × 10^8^|2.7 × 10^−7^
2	BrU0	0.9829 ± 0.1765 × 10^−1^ 0.9818 ± 0.1973 × 10^−1^	0.9470|0.9919 0.9415|0.9904	2.0 × 10^–7^ ± 9.1 × 10^−8^ 2.0 × 10^−7^ ± 9.9 × 10^−8^	1.5 × 10^−7^|3.9 × 10^−7^ 1.6 × 10^−7^|4.0 × 10^−7^
	BrU1	Phase 1			
		0.9836 ± 0.1233 × 10^−1^ 0.9849 ± 0.1346 × 10^−1^	0.9594|0.9935 0.9577|0.9935	5.6 × 10^−7^ ± 3.1 × 10^−7^ 5.4 × 10^−7^ ± 3.1 × 10^−7^	7.6 × 10^−8^|1.0 × 10^−6^ 7.8 × 10^−8^|1.0 × 10^−6^
		Phase 2			
		0.9982 ± 0.2617 × 10^−2^ 0.9954 ± 0.2146 × 10^−2^	0.9937|0.9999 0.9941|0.9992	3.6 × 10^−8^ ± 2.9 × 10^−8^ 1.1 × 10^−7^ ± 7.2 × 10^–8^	1.7 × 10^−8^|8.7 × 10^−8^ 1.3 × 10^−8^|2.0 × 10^−7^
3	BrU0	0.9919 ± 0.2000 × 10^−3^ 0.9884 ± 0.1882 × 10^−2^	0.9916|0.9921 0.9863|0.9911	1.6 × 10^−7^ ± 6.1 × 10^−8^ 1.7 × 10^−7^ ± 4.2 × 10^−8^	1.0 × 10^−7^|2.6 × 10^−7^ 1.4 × 10^−7^|2.4 × 10^−7^
	BrU1	Phase 1			
		0.9956 ± 0.5891 × 10^−3^ 0.9948 ± 0.3536 × 10^−3^	0.9948|0.9963 0.9944|0.9952	3.6 × 10^−7^ ± 2.6 × 10^−8^ 3.9 × 10^−7^ ± 1.5 × 10^−8^	3.2 × 10^−7^|3.9 × 10^−7^ 3.7 × 10^−7^|4.1 × 10^−7^
		Phase 2			
		0.9973 ± 0.3493 × 10^−3^ 0.9986 ± 0.2191 × 10^−3^	0.9967|0.9975 0.9983|0.9989	5.8 × 10^−8^ ± 2.2 × 10^−8^ 4.6 × 10^−8^ ± 1.4 × 10^−8^	3.9 × 10^−8^|9.5 × 10^−8^ 3.4 × 10^−8^|6.6 × 10^−8^

### Part 2 — Investigation of the influence of asymmetrical volume change and flow rate on gas transport

Figure 5 shows 4 of 12 simulation results of *V*_g,BrU0_(*t*), *V*_g,BrU1_(*t*) and *V*_g,BrU2_(*t*) under asymmetric conditions for BrU1 and BrU2, respectively, with a diffusion coefficient of 0.3 cm^2^/s. Table 3 show the conditions with and without convection that were used for the simulations labelled 1 through 6. The curves 1, 1′, 3, and 3′ correspond to the conditions of simulation 1, the curves 2, 2′, 4, and 4′ to the conditions of simulation 6.

**Fig. 8:**
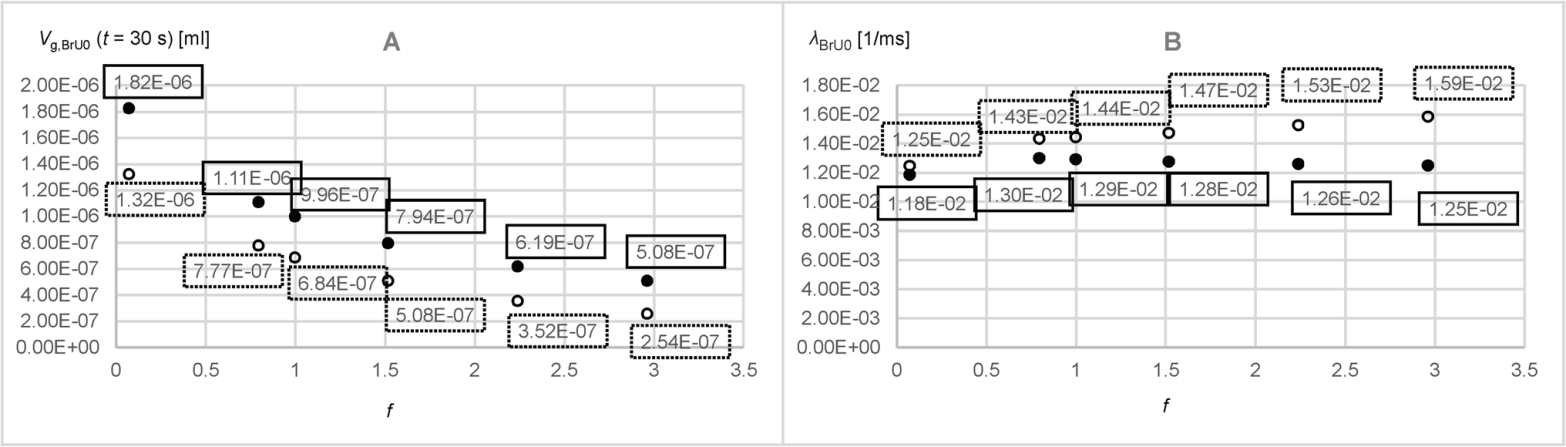
Parameters obtained from the time diagrams *V*_g,BrU0_(*t*) of Part 2 of the parameter study according to Eq. 22 for pure diffusion (filled circles) and with additional convection (unfilled circles)

**Fig. 9:**
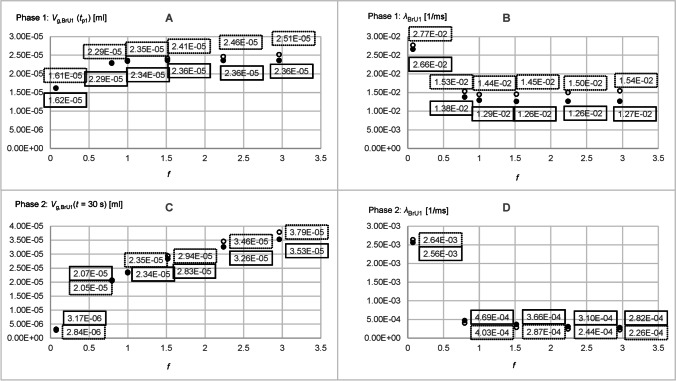
Parameters obtained from the time diagrams *V*_g,BrU1_(*t*) of Part 2 of the parameter study according to Eq. 22 for pure diffusion (filled circles) and with additional convection (unfilled circles)

Figure 5A and C show that the curves already reached their equilibrium value (Figs. 8A and 9C) according to Eq. 23 before the simulation end of 30 s, if gas transport was by diffusion only. If convection was added, more tracer gas flowed out of BrU0 as Fig. 5A reveals. As a consequence, *V*_g,BrU0_(*t* = 30 s) became smaller (Fig. 8A), while the transport process was accelerated, resulting in a greater *λ*_BrU0_(*t*) (Fig. 8B).

As can be seen from Fig. 5D, the two phases of the volume curves *V*_g,BrU1_(*t*) and *V*_g,BrU2_(*t*) used to approximate the simulated results change depending on the degree of asymmetry between the volumes *V*_BrU1_ and *V*_BrU2_. If the ratio *f* between *V*_BrU1_ and *V*_BrU2_ is less than 1 in the second phase, tracer gas flows from BrU1 to BrU2 (curves 1, 1′, 3, and 3′), whereas the opposite happens when the volume ratio *f* is greater than 1 (curves 2, 2′, 4, and 4′). In the symmetrical case where the ratio *f* is 1, there was no second phase.

For very small volumes *V*_BrU1_ of 5.76 × 10^−4^ ml, the first phase ended earlier than for larger volumes *V*_BrU1_ such as 2.3 × 10^−2^ ml (Fig. 5D), since a larger volume is associated with a longer diffusion path length and thus results in a smaller *λ* (Fig. 9B). With convection, these *λ* were increased compared to those without convection, so the tracer gas transport accelerated and the first phase ended earlier.

Figure 5C shows that the values of tracer volume *V*_g,BrU1_(*t* = 30 s) (Fig. 9C) differ from those of pure diffusion. It was found that in the range where the ratio *f* was less than 1, the values of *V*_g,BrU1_(*t* = 30 s) at the end of the simulation were below those of pure diffusion and in the range of *f* greater than 1. The opposite is true for BrU2. However, Fig. 9A reveals that the transition is not at *f* equals 1 since it depends not only on *f* but also on the absolute values of *V*_BrU1_ and *Q*_BrU1_. This was also evident in Part 1 of the study.

Gas transport between BrU1 and BrU2 in the second phase was slowed with increasing volume *V*_BrU1_ and thus increasing diffusion length (Eq. 8), resulting in a smaller *λ* (Fig. 9D).

**Fig. 10:**
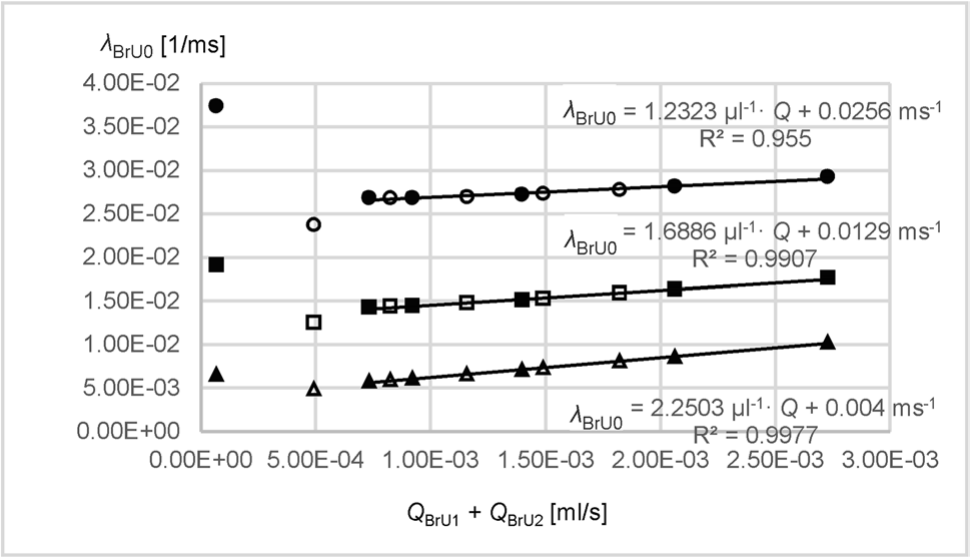
Growth constants *λ*_BrU0_ of BrU0 under the conditions of Part 1 (filled data point symbols) and Part 2 (non-filled data point symbols) for the diffusion coefficients 0.1 cm^2^/s (triangles), 0.3 cm^2^/s (rectangles), and 0.6 cm^2^/s (circles). The values *λ*_BrU0_ were plotted against the total volume flow *Q*_BrU1_ + *Q*_BrU2_ at the branching point. For all data points with volume flows above 5 ml/s, linear trend lines were calculated with Microsoft Excel 2016 and the trend line function as well as the degree of determination *R*^2^ were specified

To investigate the sensitivity of diffusion and convection to gas dynamics, simulations were carried out under the conditions of Parts 1 and 2 for the diffusion coefficients 0.1 cm^2^/s and 0.6 cm^2^/s in consideration of convection. Figure 10 shows the growth constants *λ*_BrU0_ of BrU0 under the conditions of Part 1 (filled data points) and Part 2 (non-filled data points) for the diffusion coefficients 0.1 cm^2^/s (triangles), 0.3 cm^2^/s (rectangles), and 0.6 cm^2^/s (circles). The values *λ*_BrU0_ were plotted against the total volume flow *Q*_BrU1_ + *Q*_BrU2_ at the branching point. The figure shows that for volume flows greater than 5 × 10^−4^ ml/s, the growth constants *λ*_BrU0_ increased approximately linearly with linear increasing volume flow with $\overline{{\text{R} }^{2}}$ between 95.50 and 99.77%. From the slopes of the trend lines (calculated with Microsoft Excel 2016, Microsoft, Redmond, WA) of 1.23 µl^−1^ for 0.6 cm^2^/s, 1.69 µl^−1^ for 0.3 cm^2^/s, and 2.25 µl^−1^ for 0.1 cm^2^/s, it can be deduced that gases with low diffusion coefficients such as SF_6_ are more sensitive to changes in volume flow than gases with high diffusion coefficients such as He (see Fig. 10).

Table 5 shows the mean values and standard deviations of $\overline{{\text{R} }^{2}}$ and $\overline{\text{RMSE}}$ from the curve fittings for the sensitivity analysis. They were averaged over all simulations for BrU0.

**Table 5:** Mean values and standard deviations of the coefficients of determination ($\overline{{\text{R} }^{2}}$) and root-mean-square errors ($\overline{\text{RMSE}}$) with the corresponding minimum and maximum values from the trend line determinations of *V*_g,BrU0_(*t*) for BrU0 of Parts 1 (first value) and 2 (second value) for the diffusion coefficients *D* = 0.1 cm^2^/s and *D* = 0.6 cm^2^/s

Diffusion coefficient (cm^2^/s)	$\overline{{\text{R} }^{2}}$		$\overline{\text{RMSE}}$(ml)	
	Mean ± SD	Min|max	Mean ± SD	Min|max
0.1	0.9816 ± 0.2435 × 10^−1^ 0.9853 ± 0.1626 × 10^−1^	0.9319|0.9923 0.9521|0.9921	2.6 × 10^−7^ ± 1.3 × 10^−7^ 2.8 × 10^−7^ ± 1.5 × 10^−7^	2.0 × 10^−7^|5.2 × 10^−7^ 2.0 × 10^−7^|5.9 × 10^−7^
0.6	0.9271 ± 0.1519 × 10^0^ 0.9798 ± 0.2113 × 10^−1^	0.6171|0.9893 0.9367|0.9890	1.9 × 10^−7^ ± 1.7 × 10^−7^ 1.5 × 10^−7^ ± 7.2 × 10^−8^	1.2 × 10^−7^|5.5 × 10^−7^ 1.2 × 10^−7^|3.0 × 10^−7^

### Part 3 — Investigation of the influence of changes in the diffusion coefficient under asymmetrical conditions

**Fig. 11:**
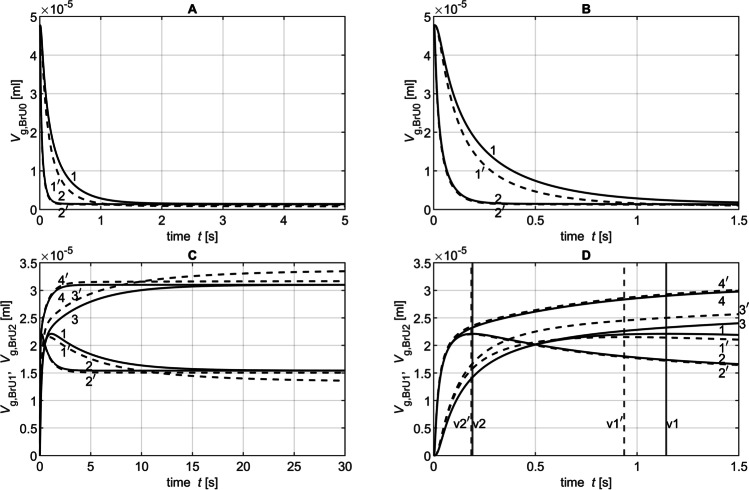
Time diagrams of *V*_g,BrU0_(*t*) (**A**, **B**), *V*_g,BrU1_(*t*) (**C**, **D** curves 1, 1′, 2 and 2′) and *V*_g,BrU2_(*t*) (**C**, **D** curves 3, 3′, 4 and 4′) from Part 3 of the parameter study with (dotted lines) and without (solid lines)convection for different time intervals. The curves 1, 1′, 3, and 3′ correspond to the conditions of simulation 1 in Table 3; the curves 2, 2′, 4, and 4′ correspond to those of simulation 5. The vertical lines v1 and v1′ mark the transition from phase 1 to phase 2 of the corresponding curves 1 and 1′. Accordingly, the vertical lines v2 and v2′ mark the phase transitions of the curves 2 and 2′

**Fig. 12:**
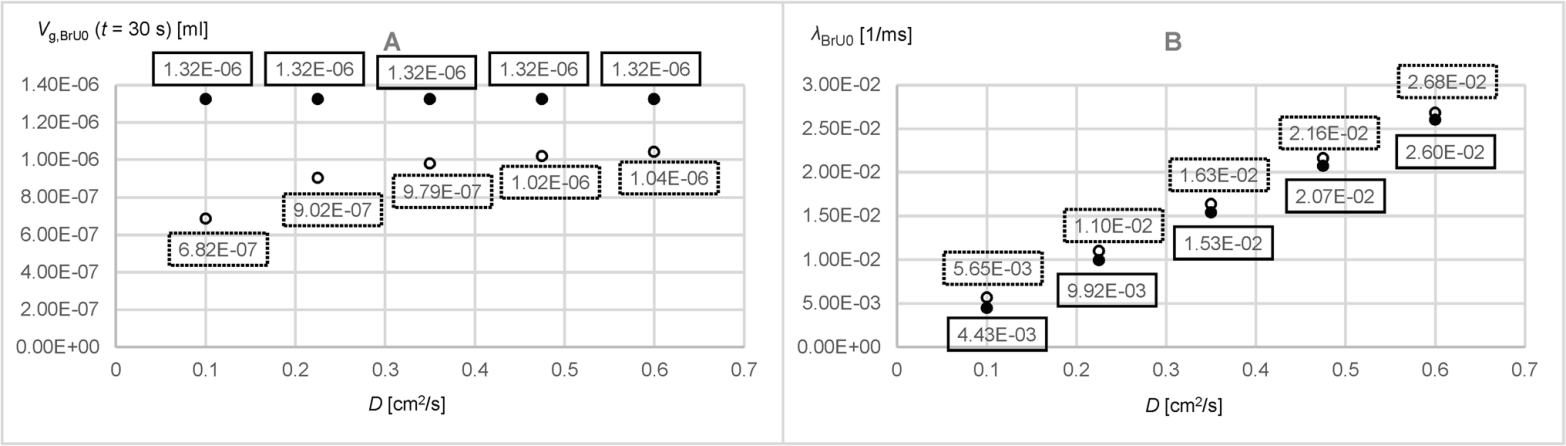
Parameters obtained from the time diagrams *V*_g,BrU0_(*t*) of Part 3 of the parameter study according to Eq. 22 for pure diffusion (filled circles) and with additional convection (unfilled circles)

**Fig. 13:**
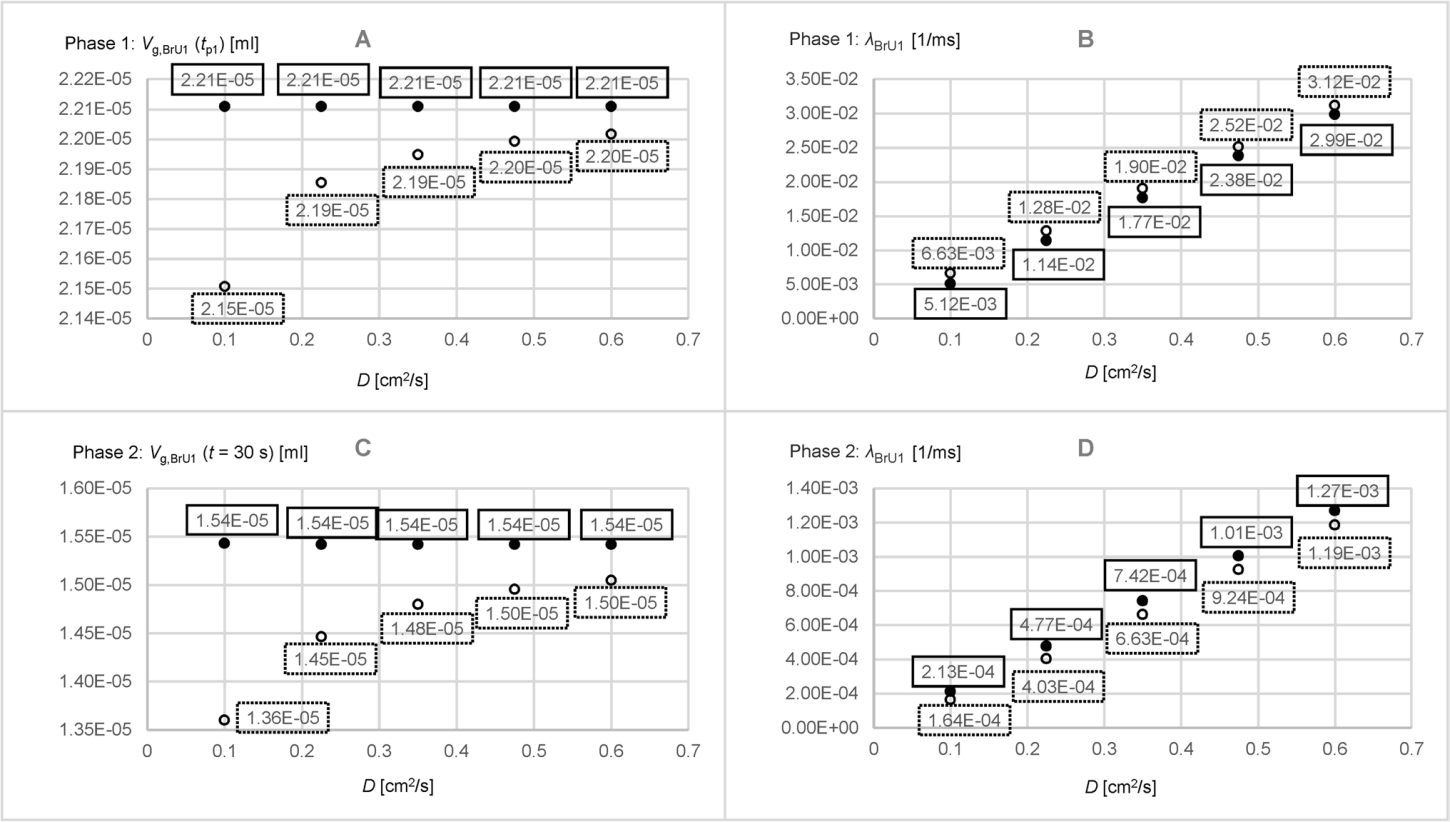
Parameters obtained from the time diagrams *V*_g,BrU1_(*t*) of Part 3 of the parameter study according to Eq. 22 for pure diffusion (filled circles) and with additional convection (unfilled circles)

Figure 11 shows *V*_g,BrU0_(*t*), *V*_g,BrU1_(*t*), and *V*_g,BrU2_(*t*) simulated with the diffusion coefficients *D* = 0.1 cm^2^/s and *D* = 0.6 cm^2^/s. These curves illustrate that in both phases, the steady state values *V*_g,BrU*i*_(*t*_p1_) and *V*_g,BrU*i*_(*t* = 30 s) for the branch units BrU0 (Fig. 12A), BrU1 (Figs. 13A, C), and BrU2 were constant and therefore independent of the diffusion coefficients when only diffusion determined the gas transport.

This is consistent with the fact that the gas in the steady state seeks an evenly distributed gas concentration, which depends only on the model geometry (Eq. 23), which was kept constant here. Thus, in a steady state, the amount of tracer gas in the first branch unit (1.5 × 10^−−5^ ml) was only half the amount of the second branch unit (3.1 × 10^−5^ ml). This ratio corresponded to the ratio of the volumes of both branch units selected for this study (Table 3). The parameters *V*_g,BrU1_(*t*_p1_) and *V*_g,BrU1_(*t* = 30 s) for both phases of BrU1 changed with an increasing diffusion coefficient, when gas transport was determined by diffusion and convection (Figs. 13A, C). The higher the diffusion coefficient, the smaller was the difference between these parameters with and without convection.

The growth constants *λ*_BrU*i*_ for the branch units BrU0 and BrU1 increased linearly with a linear increasing diffusion coefficient (Figs. 12B and 13B, D), resulting in steeper curves for *V*_g,BrU0_(*t*), *V*_g,BrU1_(*t*) and *V*_g,BrU2_(*t*) (Fig. 11).

When convection was taken into account, the growth constants are higher than with pure diffusion, which resulted in an accelerated gas transport process (Figs. 12B and 13B, D). In addition, the growth constants increased linearly with convection with an increasing diffusion coefficient.

## Discussion

In this work, a novel numerical model is presented that can be used to describe the gas transport process within the acinus via simple mathematical functions. To implement this approach, the morphometric data collected by Weibel in 1963 [34] were used to develop a numerical model of the lungs that simulates gas transport in both the bronchioles and the alveoli. The boundary conditions of the model presented in this study were set in such a way that the transport of tracer gas in the lung units could be described using simple monoexponential growth functions. As a result, the transport process was defined by only two parameters, the growth constant and steady state value. The growth constant represents the dynamics of the gas distribution while the steady-state value represents the static distribution when equilibrium is reached.

The results of this work show that the steady state values as well as the growth constants obtained from Eq. 22 mainly depend on the volume *V*_BrU*i*_ of the BrUs and the diffusion coefficient *D*. Therefore, gas transport within the acinus is mainly determined by the interactions between geometry and diffusion. This is of clinical relevance since obstructive diseases lead to anatomical changes [8] and thus the diffusive gas transport is affected.

Paiva et al. [26] suggested a two trumpet model consisting of one mother and two trumpet-shaped daughter branches linked together. The authors were able to show how the molecular diffusion properties of the gases influenced gas transport at branches in lung regions where diffusion plays a dominant role in gas transport. More recently, Whitfield et al. [27] presented a new approach to investigate the influence of the asymmetry in a bronchial tree on ventilation inhomogeneities and thus on the parameters determined with MBW. The model developed by Hasler et al. [28] considered both the asymmetric structure of the air conducting airways and the compliance of the peripheral airways and alveoli. However, the asymmetry of the peripheral airways was not considered since part of the air-conducting and peripheral airways were combined to trumpet-shaped elements for the sake of simplicity.

The anatomical structure of the numerical model presented in this work consists of two BrUs connected in parallel, where both could be considered as dichotomous branching networks of airways. Weibel’s morphometric data [34], which could be applied to such symmetrical models, were used for the dimensioning of these airways.

Lung anatomy corresponds to an asymmetrical bronchial tree branched over several generations. In the model presented in this study, it was assumed that all bronchioles have the same length, diameter, and number of alveoli. However, in lung regions where diffusion dominates gas transport, gas distribution is particularly dependent on structural asymmetry [15]. Consequently, the assumptions made here do not reproduce the complex gas distribution mechanisms in an asymmetric airway network spanning several generations [29]. This is a limitation of the model. Nevertheless, in the model approach presented here, asymmetric conditions between BrUs were realized via different numbers of BrEs connected in series to form a BrU. In contrast to other models [26, 37], this has the advantage that by using the same lung parameters for the bronchioles, there is no cross-sensitivity between these parameters.

Although the volume flow in the individual lung alveoli varies in the lung due to the regionally different compliance [38] of the pulmonary parenchyma. A constant flow into the alveoli was assumed for the sensitivity analysis. This results in an even increase in volume over time, which is the same for all alveoli. However, the use of a constant volume flow is not a limitation of the model. It is also possible to use volume flows that change over time. In fact, the alveoli are densely packed and connected together by their walls [34], so that the air spaces cannot move independently [29]. Moreover, since diffusion processes predominate in the acinus [24], a uniform compliance of the alveoli was implemented.

In this study, the model’s ability to simulate transport processes crucial for the distribution of gas in the lungs, such as diffusion, convection, and the Pendelluft phenomenon, was investigated. The focus was on crucial parameters such as the diffusion coefficient, volumetric flow rate and geometric variables, and the evaluation of their influence on the gas distribution within the acinus. For this reason, specific boundary conditions were set to perform a parameter study deviating from medical protocols. The amount of tracer gas was kept constant, and a simulation time was chosen which was long enough to reach an almost steady state concentration value. However, this isolated examination of the transport processes in the acinus does not take into account the gas transport in the conducting airways, where convection predominates, and the convection-dependent inhomogeneities that occur there [15]. But that was not the focus of this work, where the processes in the acinus were of interest. In addition, expiration was not considered. Nevertheless, the gas distribution during normal breathing, including inspiration and expiration, depends on the breathing frequency, the tidal volume, and the signal form [39].

Despite the simplifications applied, the model presented was able to simulate, under asymmetric conditions, the diffusive Pendelluft phenomenon described by Paiva and Engel [26], in which gas exchange occurs between two lung units connected in parallel with different tracer gas concentrations.

It was also found that the amount and duration of gas transport in an asymmetric model depend not only on the degree of asymmetry, but also on the volumes and lengths of the branch units, which influence their capability to store tracer gas.

For example, both BrUs might have an extreme volume ratio and therefore a high degree of asymmetry, although the volume and thus the length of the smaller unit of the lung could be very small. In this case, however, the smaller unit can store only a very small amount of tracer gas, so the growth rates for both phases are very high, leading to a rapid achievement of steady state (Figs. 5 and 9D).

This is in agreement with the results of Paiva and Engel [37], who showed in his analyses that small units of the lung contribute only slightly to the inhomogeneities of the ventilation. Larger lung units can store much more tracer gas. Due to the longer branch unit lengths, the gas transport between the parallel units takes longer.

The analyses showed that within the acinus, the distribution of tracer gases with low diffusion coefficients like SF_6_ (Fig. 11) was more sensitive to changes in volume flow than those with higher diffusion coefficients like He (Figs. 10 and 11). This is consistent with the results of Engel et al., who had shown in their study [40] that gases with different diffusion properties distribute themselves differently within the acinus. He found that SF_6_ follows the convective flow into much deeper regions of the acinus than He.

The simulation curves could only be approximated to a limited extent via monoexponential functions. Also, the plateau at the beginning of the simulation curves (Fig. 6B, D) was not taken into account when using Eq. 17 to determine *λ*. Moreover, the separate analysis of phases 1 and 2 were sometimes difficult, especially when *V*_g,BrU*i*_(*t*) was a monotonically increasing function. In this case, the two phases could not be clearly distinguished. However, the values for $\overline{{\text{R} }^{2}}$ given in the Tables 4 and 5 show that the presented method gives sufficiently good results with two exceptions. A possible reason for these exceptions may be that a steady state had not yet been reached after 30 s (Fig. 7A, C; Tables 4 and 5).

In the model presented in this paper, the gas exchange and the adsorption and absorption processes at the mucous membranes were not taken into account. However, in the later phases of the nitrogen MBW test, N_2_ re-diffusion from the blood into the alveolar space occurs [41], though it is currently unclear what influence these processes have on the results of the washout tests [8, 42]. The Thiele modulus and, accordingly, the Damköhler number could be used in future works to assess the influence of tracer gas absorption and desorption in relation to the diffusive transport. The basic structure of the presented model had already been designed in such a way that the influences of gas exchange as well as adsorption and absorption processes on the gas distribution can be examined. For this purpose, Eq. 20 has to be extended, assuming that these processes occur in the alveolar compartments.

In this work, only the inspiration was considered in order to analyze the tracer gas transport up to the achievement of steady state values. The analysis of gas transport during a breathing cycle, the inhalation of which takes place under the boundary conditions described here, and the analysis of gas composition during the subsequent breathing cycle with variations in respiratory frequency is of clinical interest. Moreover, adsorption and adsorption processes were not modelled in this study. These aspects will be examined in more detail in further work.

## Conclusion

In this paper, a parameter study was presented to analyze the dynamics of gas transport in a simplified lung model based on the anatomical lung model by Weibel [34]. The results show that the model was able to simulate important transport processes like diffusion, convection, and the Pendelluft phenomenon. In addition, the results reveal that the molecular diffusion properties as well as the geometric conditions of the lung regions in correlation with asymmetry and diffusion path lengths have a much greater influence than convection on the dynamics of gas transport. Therefore, gas transport within the acinus is mainly determined by the interactions between geometry and diffusion, which is clinically relevant since obstructive diseases lead to anatomical changes [8] and thus influence diffusive gas transport.

## General abbreviations

BrE: Branch element
BrU: Branch unit
IGW: Inert gas washout
MBW: Multi breath washout
OLD: Obstructive lung (pulmonary) diseases
COPD: Chronic obstructive pulmonary disease
SBW: Single breath washout
VI: Ventilation inhomogeneities

## Chemical abbreviations

He: Helium
N_2_: Nitrogen
SF_6_: Sulphur hexafluoride

## Abbreviations for speed indications

*D * cm^2^/s: molecular diffusion coefficient
*Q*_Alv_ cm^3^/s: volume flow of an alveolus
*Q*_BrU__*i*_ cm^3^/s: volume flow at the input of the BrU*i*
*Q*_*k*__,in_, *Q*_*k*__,out_ cm^3^/s: volume flows at the input and output of *k*
*Q*_*k*__*’*_ cm^3^/s: volume flow in *k’*
*Q*_M_ cm^3^/s: mean volume flow
*Q*_T_ cm^3^/s: volume flow in the trachea
*u* cm/s: velocity
*u*_M_ cm/s: mean velocity

## Geometrical and anatomical abbreviations

*A*_*k*__*’*_ cm^2^: surrounding surface of the compartment *k*
*A*_*k*__,in_, * A*_*k*__,out_ cm^2^: cross-sectional areas at the input and output of *k*
*d*_Alv_ cm: diameter of an alveolus
*d*_M_ cm: mean diameter
*l* cm: length
*l*_BrU__*i*_ cm: total length of a BrU*i*
*l*_M_ cm: mean length
*m*_Alv_: number of alveoli per bronchiole
${\stackrel{\mathrm{-}}{m}}_{\text{Alv}}$: averaged number of alveoli
*m*_Alv,BrU__*i*_: total number of alveoli in each BrU*i*
*m*_Br_: number of bronchioles
*r*_*k*_: cm radius of *k*
*V*_Alv_ cm^3^: volume of an alveolus
*V*_BrU__*i*_ cm^3^: volume of a BrU*i*
*V*_BrU__*i*__,__*k*_, *V*_BrU__*i*__,__*k*_ cm^3^: volumes of *k* and *k’* within a BrU*i*
*V*_*k*_, *V*_*k'*_ cm^3^: volumes of *k* and *k’*
*V*_ref_ cm^3^: reference volume
*V*_tot_ cm^3^: total volume (sum of the volumes of all BrUs)
*x*_*i*_ cm: axis of a BrU*i*
Δ*x * μM: local resolution
*z*: generation

## Molar and gas specific abbreviations

*c*_*k*_, * c*_*k'*_ mol/m^3^: concentrations in *k* and *k’*
*n*_*k*_, * n*_*k'*_ mol: molar quantities in *k* and *k’*
$\dot{n}_{k\prime }$ mol/s: molar flux in *k’*
$\dot{n}_{{k,{\text{in}}}}$, $\dot{n}_{{k,{\text{out}}}}$ mol/s: inflowing and outflowing molar fluxes of *k*
*V*_g__,BrU__*i*_ cm^3^: volume of tracer gas in BrU*i*
*V*_g__,Tr_ cm^3^: total volume of tracer gas
*V*_g__,st,BrU__*i*_ cm^3^: volume of tracer gas at steady state within a BrU*i*
V_m_ m^3^/mol: molar volume
*χ*_*k*_, *χ*_*k*__*’*_: mole fractions in *k* and *k’*
*χ*_BrU__*i*__,__*k*_, *χ*_BrU__*i*__,__*k*__*’*_: mole fractions in *k* and *k’* within BrU*i*

## Temporal abbreviations

*t* s: time
*t*_p1_ s: time at the end of phase 1 of *V*_g,BrU__*i*_(*t*)
*t*_start_, * t*_end_ s: start and end time of phases 1 and 2 of *V*_g,BrU__*i*_(*t*)
*λ*_BrU__*i*_ 1/ms: growth constant of *V*_g,BrU__*i*_(*t*) for phase 1 and 2 respectively

## Statistical abbreviations

${\text{R} }^{2}$: Coefficient of determination
$\text{RMSE}$ cm^3^: Root-mean-square error
